# Semaphorin 7a is a biomarker for recurrence in postpartum breast cancer

**DOI:** 10.1038/s41523-020-00198-1

**Published:** 2020-10-19

**Authors:** Virginia F. Borges, Junxiao Hu, Chloe Young, Jaron Maggard, Hannah J. Parris, Dexiang Gao, Traci R. Lyons

**Affiliations:** 1 Young Women’s Breast Cancer Translational Program, University of Colorado Cancer Center, Aurora, CO USA; 2 Division of Medical Oncology, University of Colorado, Anschutz Medical Center, Aurora, CO USA; 3 Department of Pediatrics, School of Medicine, and Dept of Biostatistics, University of Colorado School of Public Health, Aurora, CO USA; 4 Department of Epidemiology, University of Colorado School of Public Health, Aurora, CO USA

**Keywords:** Breast cancer, Prognostic markers

## Abstract

Breast cancer is a global health threat and cases diagnosed in women during the years after childbirth, or postpartum breast cancers (PPBCs), have high risk for metastasis. In preclinical murine models, semaphorin 7a (SEMA7A) drives the metastatic potential of postpartum mammary tumors. Thus, we hypothesize that SEMA7A may drive metastasis of PPBC in women. We report that SEMA7A protein expression is increased in PPBCs compared to their nulliparous counterparts in our University of Colorado cohort. Additionally, tumors from PPBC patients with involved lymph nodes and lymphovascular invasion were higher on average suggesting a potential role for SEMA7A as a prognostic biomarker. Consistent with this hypothesis we identify a level of SEMA7A expression in tumors that can predict for recurrence. We propose SEMA7A as a potential biomarker and therapeutic target for PPBC patients, who currently lack strong predictors of outcome and unique targeted therapy options.

## Introduction

Breast cancer is a global health threat with an estimated 1.7 million cases annually. Death due to breast cancer disproportionately affects low income countries and younger women in these countries are particularly at risk^1^. In the United States, about 27,000 younger women are affected by breast cancer annually, with age <35 and diagnosis within ten years of most recent childbirth as two unique risk factors for breast cancer metastasis and death^2–4^.

Prior to 2009, pregnancy-associated breast cancer (PABC) was defined as any breast cancer diagnosed during pregnancy or in the 1–2 years immediately after childbirth^5^. However, significant research has clarified that women diagnosed during pregnancy fare equal to their non-pregnant peers with similar breast cancers^2,6,7^. Conversely, women diagnosed in the early postpartum years face significant increased risk for metastasis in comparison to nulliparous women or women whose children are older, despite their breast cancers having similar risk profiles. Therefore, it is important to separate PABC, especially those diagnosed during pregnancy, from postpartum breast cancer (PPBC) as the outcomes are clearly different^2,3,5,8^.

PPBC is currently defined as a breast cancer diagnosis occurring within ten years following childbirth^3,8^. In one large epidemiology study, women diagnosed with PPBC experienced a 2-fold increase in mortality risk when diagnosed 4–6 years following childbirth, while women within two years postpartum experienced a thirty percent increase in mortality rate^2^. In more recent studies where detailed tumor characteristics were available, women diagnosed within 5 years of giving birth had a 3-fold risk of metastatic recurrence without a significant difference present in the clinical risk characteristics of the tumors, as compared to nulliparous or women whose most recent childbirth occurred more than 10 years before diagnosis^8^. This increased risk period was then extended when a larger cohort study demonstrated that postpartum women diagnosed with Stage 1 and 2 tumors up to 10 years from their most recent childbirth were at a 3–5 fold significantly higher risk for developing metastases, with tumors continuing to recur up to 15 years after diagnosis^4^. In these studies, a postpartum diagnosis was associated with a higher incidence of having lymphovascular invasion (LVI) and involved lymph nodes (LN)^4^.

Our group has recently reported that a novel protein may be implicated in PPBC in preclinical models^9–12^. Semaphorin 7a (SEMA7A) is a GPI-linked member of the Semaphorin family of signaling proteins. SEMA7A is involved in many normal and diseased biological processes^13–15^. We previously reported from available genomic databases that *SEMA7A* mRNA expression is increased and associated with worse prognosis in breast cancer. Also, we have shown that increased SEMA7A protein expression promotes tumor growth, motility, invasion, and lymphangiogenesis^9–11^. Finally, in a small subset of postpartum women we observed increased expression of SEMA7A in their normal breast tissue^12^.

In this pilot study, we sought to investigate the role of SEMA7A expression in young women’s breast cancer (YWBC). We performed a matched study to determine the relationship between human PPBC and SEMA7A expression, and how SEMA7A is correlated with PPBC and clinically significant risk factors and outcomes. We believe our results may provide the basis for further investigation of SEMA7A in PPBC for the development of novel prognostic and therapeutic tools, using SEMA7A as a biomarker, in order to better care for PPBC patients.

## Results

We included 113 cases from our Colorado Young Women’s Breast Cancer Cohort where we published an increased risk for metastatic recurrence in PPBC^4^. There were 47 nulliparous patients and 66 PPBC patients that made it into the study (Supplementary Table 1). Our cases averaged 36 years of age with a range of 26–45. Overall, our groups were similar in age, race, stage, tumor size, grade, histology, biologic subtype, LN, and LVI. All PPBC patients were diagnosed within 5 years of a completed pregnancy with 38 patients (57.6%) diagnosed within 2 years and the remaining 28 (42.4%) diagnosed greater than 2 and less than five years from most recent childbirth. Among our PPBC cases, 19 (28.8%) were uniparous and 46 (69.6%) were multiparous, range 1–4 children. One case had at least one child without data on total number of children and three cases had complete parity information but unknown total number of pregnancies (Supplementary Table 2). All cases had invasive disease present and 39 (77%) contained adequate matched normal-adjacent breast tissue on the same tissue section. We intentionally aimed for a similar number of cases with and without disease recurrence between our nulliparous and PPBC groups in our design. Based on available cases with tissue, this resulted in our nulliparous cases having 27 (57.5%) without and 20 (42.5%) with disease recurrence, distant and local-regional combined. For the PPBC cases, 42 (63.6%) with no recurrence, 19 with recurrence (28.8%), 4 other (6.06%), and 1 missing (1.5%) (Supplementary Table 3).

### SEMA7A expression, breast cancer clinical characteristics, and parity status

**Fig. 1:**
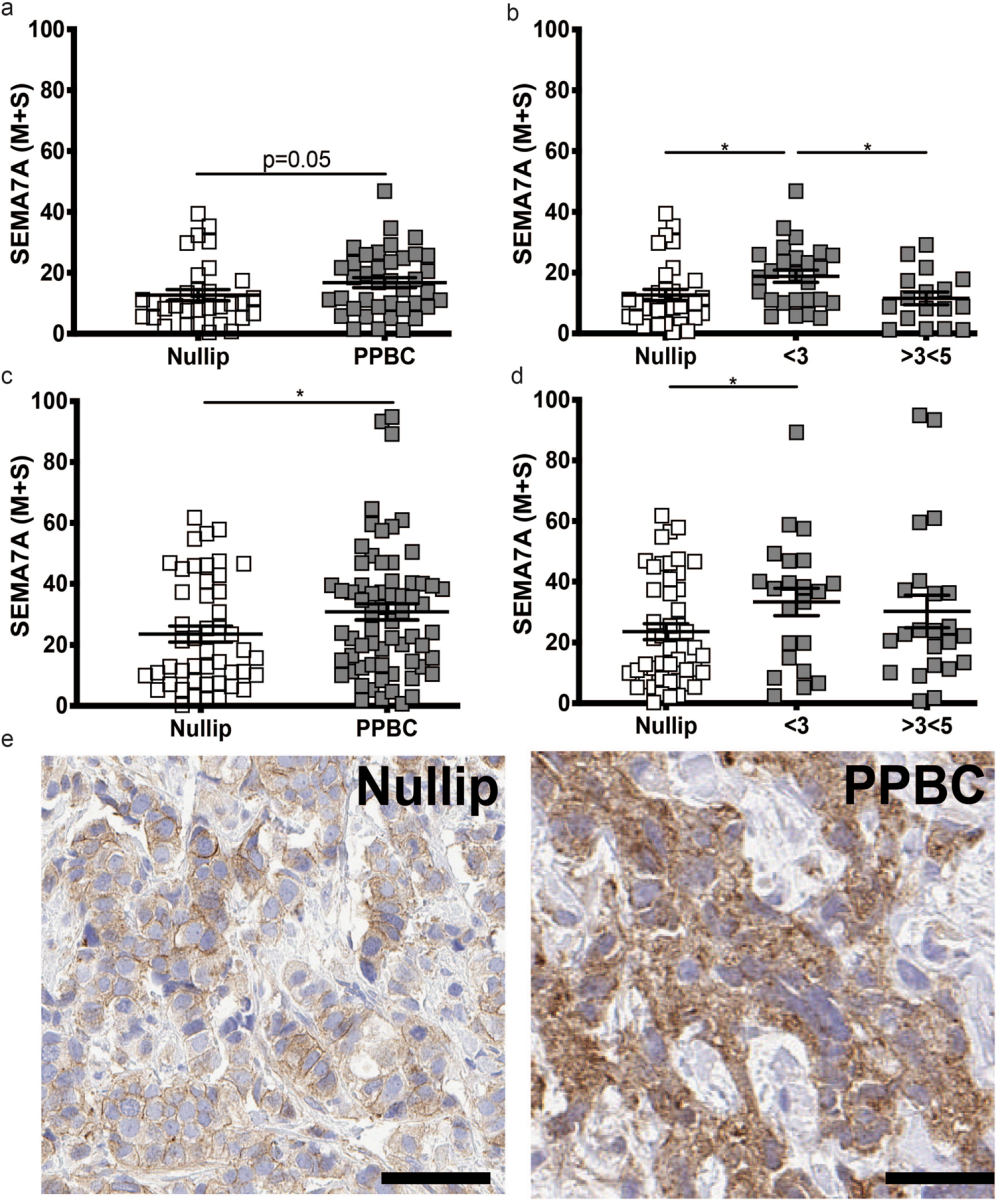
Semaphorin 7a expression is increased in normal and breast tumor tissues from PPBC. **a** % SEMA7A medium + strong (M + S) in normal-adjacent breast tissues from women who were nulliparous (Nullip) or within 5 years of last childbirth (PPBC) at the time of tissue collection. **b** Data from **a** stratified by years since last childbirth. **c** % SEMA7A medium + strong (M + S) in breast tumor tissues. **d** Data from **c** separated by years since last childbirth. *p < 0.05, one-tailed Student’s *t*-test, mean + SEM are presented. **e** Representative images from Nullip and PPBC cases stained for SEMA7A. Scale bar = 50 uM.

**Fig. 2:**
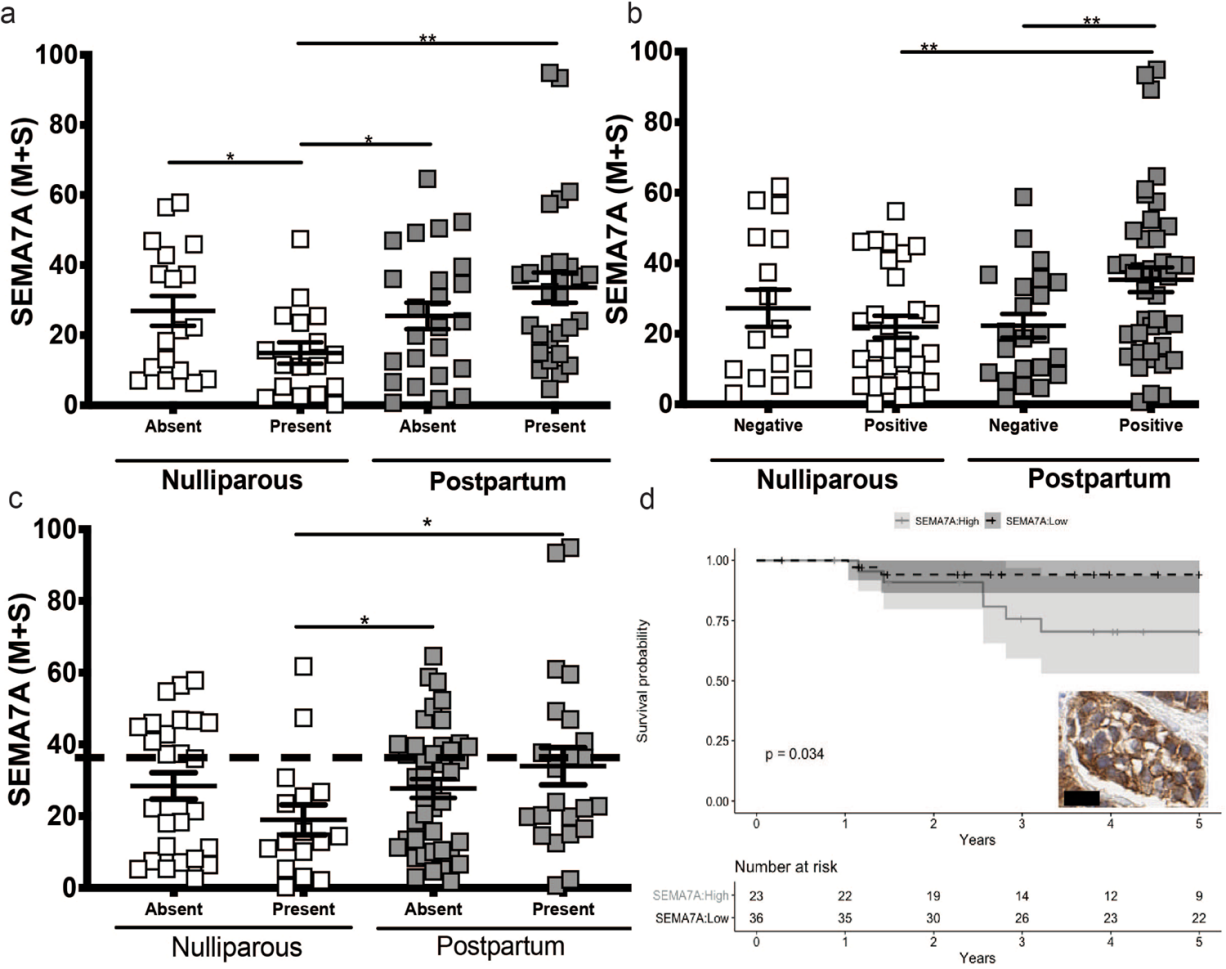
Semaphorin 7a expression drives poor outcomes in PPBC. SEMA7A (M + S) staining in patients stratified by **a** Lymphovascular invasion, **b** Lymph node involvement, and **c** Recurrence status. **d** Dichotomization of PPBC with an optimal cutoff of 36% M + S for SEMA7A high significantly predicts for decreased recurrence-free survival. (inset) PPBC case that is 36% SEMA7A + . Scale bar = 10 um. *p < 0.05, **p < 0.01, one-tailed Student’s *t*-test, mean + SEM are presented.

Using an antibody raised against amino acids 371–441 in the SEMA7A protein sequence, which will recognize all isoforms of SEMA7A due to sequence homology, we compared SEMA7A expression with multiple breast cancer clinical characteristics that are known prognostic indicators across all 113 cases. There were no differences seen in SEMA7A expression based on grade, stage, LN status, LVI, or biologic subtype when cases were analyzed as one combined group (Supplementary Fig. 1). We then analyzed the expression of SEMA7A between the nulliparous and PPBC groups. First, we observed that SEMA7A expression was significantly increased in the normal-adjacent tissues of the PPBC group compared with nulliparous and that this increase is most notable for those cases diagnosed within 3 years of last pregnancy and declines among cases that are further out from their last childbirth with significance lost by a diagnosis >3 to <5 years after birth (Fig. 1a, b). We similarly show that PPBCs have higher tumor SEMA7A expression than nulliparous, however, in the tumors the level of SEMA7A expression remains higher even among cases with more time between last parturition and diagnosis, without a decrease as seen in the normal tissue, up to our 5 year current cutoff (Fig. 2c, d). Of note, ten cases in the nulliparous group had previously been pregnant once (8) or twice (2), yet removal of these cases from the analysis did not change the overall result. Additionally, total parity number did not correlate with any significant difference in SEMA7A expression among PPBCs (Supplementary Fig. 2a, b).

Focusing further on the tumor analysis, SEMA7A expression did not differ between nulliparous and PPBC groups when compared for grade, stage, or biologic subtype (Supplementary Fig. 3A–C). There was, however, a significant difference seen in the level of SEMA7A expression in PPBC cases that had evidence of lymphogenous spread. Specifically, postpartum patients with LN positivity exhibited elevated levels of SEMA7A compared to LN-positive nulliparous patients. Similarly, SEMA7A expression was significantly higher in PPBC patients with evidence of LVI when compared to nulliparous (Fig. 2a, b). Lastly, we evaluated SEMA7A expression between cases with and without breast cancer recurrence in our nulliparous and PPBC groups and found that high SEMA7A expression correlated with poorer outcomes in PPBC, but not in nulliparous. Specifically, SEMA7A expression was observed to be highest in PPBC patients that had subsequently faced a breast cancer recurrence with local-regional or metastatic disease (Fig. 2c; Supplementary Fig. 4). Of note, SEMA7A was actually significantly lower in nulliparous patients with recurrence when compared to PPBC regardless of recurrence status. Thus, we performed Kaplan–Meier analysis to determine a level of SEMA7A that significantly associated with increased probability of recurrence in the PPBC group only. We were able to include 59 PPBC patients. Less than 50% of the patients died during the follow-up period, thus, the median survival was not reached. We determined that stratification of patients based on ~36% SEMA7A positivity, defined as at least 36.1% positive pixels by quantitative IHC, generated recurrence-free survival curves that are statistically significant different between the SEM7A high and low groups for PPBC patients (*p* = 0.03) with 23 patients categorized into the SEMA7A high group and 36 patients in the SEMA7A low group (Fig. 2d; Supplemenatry Table 4). These results provide impetus for further exploration of SEMA7A and recurrance free survival (RFS) in a larger cohort of young women with breast cancer.

## Discussion

Postpartum breast cancer is a unique subset of breast cancers that represent a leading cause of cancer-related deaths in young women. However, despite the worsened outcomes associated with PPBC, there are no targeted therapies available for these patients. The recommended management of care for patients with PPBC is simply to follow current treatment guidelines for premenopausal breast cancer^16^, which has contributed to the growing disparity of poor prognosis in YWBC^1^. Further investigations into the mechanisms behind the aggressive nature of postpartum breast cancer are crucial, as we should be able to eradicate the poorer rates of breast cancer free survival in postpartum women.

To understand the underlying molecular basis behind the intensity of PPBC, our study sought to validate our preclinical studies suggesting a role for SEMA7A in human PPBC^17^. We have now identified SEMA7A as a potential biomarker for prognosis and a target for intentional therapy against PPBC. The specific increase in SEMA7A expression in the normal-adjacent tissue in our PPBC cases also supports our prior preclinical work that demonstrated SEMA7A is involved in postpartum involution, the normal process of postpregnancy/postlactation return of the breast to a non-secretory epithelium. A study seeking to investigate gene expression alterations during involution found that, among other immune-related genes, SEMA7A gene expression was in fact present during this process^18^. It has also been shown that while SEMA7A expression is rare in nulliparous murine mammary epithelium, postpartum mammary epithelium resulted in ~8–15% of the epithelial cell population expressing SEMA7A on day 6 of involution^12^. We believe these findings are significant to human PPBC because in mouse models postpartum involution has been shown to increase the aggressiveness of breast cancers arising during the postpartum/postlactation window^10–12,19^. The durability to the elevation of SEMA7A expression in the normal breast in our cases that were diagnosed up to 3 years postpartum offers understanding of how a pathway upregulated during involution could alter prognosis of a cancer that arises during this moment yet is diagnosed years later.

Most importantly, we have found that SEMA7A expression is significantly increased in invasive breast tumors of postpartum women, with a durable increase seen in cases diagnosed up to at least 5 years from last childbirth. This indicates a role for SEMA7A overexpression in the normal-adjacent postpartum breast leading to a commensurate enduring overexpression of SEMA7A in the developing breast cancers. In this study, SEMA7A expression does not correlate with typical breast cancer risk factors, such as stage, grade, ER expression, or biologic subtype, suggesting it is independent of these typical prognostic factors and consistent with our published data that the poorer prognosis of PPBC is not associated with these factors^4,8^. Conversely, SEMA7A expression does correlate with LN positive and LVI positivity in our PPBC cases, which aligns with our published data on increased LN and LVI in PPBC compared with nulliparous or later parous [more than 10 years postpartum at diagnosis] YWBC cases, which have better prognosis than PPBC^8^. Also, in this matched study, where the presence or absence of recurrence was equivalent across experimental groups, SEMA7A was highest in our PPBC cases with breast cancer recurrence and we identified a cut point of 36% positivity for SEMA7A that could predict for recurrence by Kaplan–Meier survival statistics. Collectively, these data support the hypothesis that it is not the tumor centric characteristics of proliferation measures that influence PPBC outcome, but something related to lymphatic invasion and lymphogenous metastasis, with SEMA7A appearing significantly involved.

The role of postpartum involution as causative to the poorer prognosis of breast cancer arising in the postpartum window was first proposed by Dr. Pepper Schedin^20^. Extensive preclinical and translational research has proven that the involution process involves intrinsic tissue-remodeling pathways that are essential for dedifferentiating the mammary gland back to the prepregnant state. It has been found that this involution process involves an immunological program that mimics wound healing^19^, increased recruitment of suppressive macrophages and fibrillar collagen deposition^18^ and lymphangiogenesis^11^. Notably, this increased collagen deposition has also been observed to consequently increase COX-2 expression, which promotes lymphangiogenesis and LN metastasis in tumors present in the involuting mammary gland^10,11^. We have shown that COX-2 expression in the breast cancer cells correlates with SEMA7A expression, which is then both necessary and sufficient for the resultant increase in lymphangiogenesis^9^. SEMA7A also promotes the macrophages present during involution that remodel into novel lymphatics, increasing lymph vessel density in the involuting mammary gland and enhancing lymphatic spread of breast cancer^9^. Additionally, SEMA7A is necessary for the collagen and COX-2 deposition observed in preclinical models of PPBC and SEMA7A expression in tumors in nulliparous hosts is sufficient to drive these same characteristics^17^. These data, together with our current study’s data, link the role of SEMA7A as increasing during postpartum involution, causing a normal increase in protumorigenic changes, including lymphatic channels in the remodeling breast, and also resulting in the overexpression of SEMA7A in developing breast cancers with increased propensity towards LVI and LN positivity at breast diagnosis in postpartum women. We suggest that it is through this mechanism, that increased SEMA7A expressing breast cancers would then have a durable higher risk for recurrence and worse outcomes.

Our study is limited by the small matched method used, and these results will be validated in a larger cohort study where greater detail of the relationship between SEMA7A expression, different biologic subtypes, expanded age and parity groups, and attention to other important prognostic characteristics, such as race, can be explored. Our recent results indicate that in our Young Women’s Colorado Cohort there were more luminal B cancers than luminal A, with the expected poorer prognosis. Our current results show that, when separated by subtype, the highest percentage of PPBC cases that are SEMA7A high, 58%, is in the luminal B subtype. Thus, the role of SEMA7A in luminal B cancers should be explored. Importantly, our cohort is limited by inclusion of predominantly white women with insurance coverage and normal BMI’s, reflecting the demographics of our institution and state. We believe this likely results in our data looking more favorable [less recurrences] than a more diverse cohort that reflects the global picture of YWBC. Thus, we will expand future studies to additional cohorts with more diverse patient populations through collaboration with other institutions.

In summary, we present the first results of SEMA7A expression linked with increased breast cancer recurrence in PPBC and highlight that this association tracks specifically with LVI and LN positivity at diagnosis. Our data suggest a mechanism linked through the increase of SEMA7A mediated lymphangiogenesis occurring during normal postpartum breast involution that can durably affect breast cancers arising in the subsequent 5 years after childbirth and associates with the unique poorer outcomes of PPBC. Further study into SEMA7A as a prognostic biomarker and potential therapeutic target for this deadly subset of breast cancer are warranted and ongoing.

## Materials and methods

### Study approval

The clinical trials under which all data and human tissues were collected for this research were conducted according to Declaration of Helsinki Principles and with approval of the Colorado Multiple Institution Review Board. Subjects were enrolled before 2010 through a retrospective consent exempt protocol and after 2010 they were prospectively enrolled and provided written informed consent for participation.

### Subject selection criteria

The Colorado Young Women’s Breast Cancer Cohort includes a large database of breast cancer cases with a subset having tissue obtained from clinically indicated core biopsies or surgical procedures. For this database, all clinical data were originally collected from the patient’s medical record with outcomes obtained from our tumor registry q 6 months. Pathologic characteristics are collected directly from the original pathology reports. Race is a self-reported variable obtained from the patient at the time of first visit to the institution. Eligibility for the main protocols include: ≤45 years of age at diagnosis, availability of parity data, acceptance of recommended treatment for curative intent cases, and follow-up for breast cancer recurrence. The data are stored in RedCap™. To compare the SEMA7A levels between the patients’ groups of interest, we used a matched design with parity being the main criteria of interest, as follows: nulliparous with and without breast cancer recurrence compared to PPBC with and without breast cancer recurrence, as our main experimental groups. All patients were premenopausal at the time of diagnosis. Nulliparous cases were defined as never pregnant (G0P0) or without completed childbirth (GXP0), where X may be any number, as we have previously published no difference in outcomes among women with prior incomplete pregnancies (GXP0, X = 1 or more) and breast cancer recurrence^8^. PPBC cases were defined as having completed at least one childbirth (GXPX), where X = 1 or more, and having received a cancer diagnosis within 5 years of parturition. Given the strong influence biologic subtype has on breast cancer recurrence, we intentionally aimed to select ~70% to be ER positive and 30% to be TNBC (ER/PR/Her2 negative) between nulliparous and PPBC cases. Only Her2-negative cases were included for simplicity in this pilot study to eliminate confounding variables of crosstalk from overlapping signaling pathways. Lastly, we selected a random sample of the identified cases that had available tumor sections, which included normal-adjacent tissue. Additional data on clinically significant variables were available as follows: histologic subtype (ductal, lobular, inflammatory, other), biologic subtype (luminal A versus B determined as estrogen receptor positive and progesterone receptor positive/A or negative/B and centralized Ki-67 proliferation index testing as <14% low/A or ≥14% high/B respectively), stage (AJCC version 7 TNM criteria), lymph node involvement (LN), lymphovascular invasion (LVI), grade (from clinical pathology report), and breast cancer recurrence (absent or present, where present includes local-regional recurrence, metastatic disease or both). Other status (for recurrence local/regional/metastatic) is a combination of when a subject had either “recurrence of unknown type” or “patient stage IV never free of disease”.

### Immunohistochemistry

REMARK guidelines were followed for our study. For Tissues were formalin fixed and paraffin embedded as previously described^18^ and stored at 4 °C. Four micrometer sections of paraffin-embedded human tissue were deparaffinized and pretreated with 1X Dako Target Retrieval solution under pressure for 5 min. Slides were prepared in a Dako Autostainer using semaphorin 7a primary antibody (SEMA7A C-6, Santa Cruz) at a 1:500 dilution. Immunoreactivity was detected using Envision+ Mouse secondary antibody (Dako). 3,3’-diaminobenzidine was used as the chromogen (Dako, 10 min). Hematoxylin was used as the counterstain (Dako, 6 min). The majority (>80%) of cases had Ki-67% index staining centrally performed as previously described^4^. Staining was done in multiple batches with the same control stained each time and utilized to normalize for batch to batch variation. The same lot of antibody was utilized for all staining.

Staining quantification was done using Aperio ImageScope software (Leica Biosystems). Histological sections were digitally scanned using Aperio ScanScope3 equipment. Each section was assessed for normal-adjacent tissue and invasive breast cancer (IBC), and peritumoral lymphatic vessel density in a blinded manner. Tissues were subsequently annotated for representative regions of each tissue category present. Annotated regions were analyzed for percent positive staining using a proprietary color deconvolution algorithm created in ImageScope. Percent positive was calculated as the sum of total medium and strong (M + S) positive signal, divided by the total annotation area, and multiplied by 100.

### Statistical analysis

Two-sample independent *t*-tests were used to compare the distributions of the continuous outcomes between Nullip vs PPBC groups, and the paired comparisons between subgroups; Fisher’s exact tests were conducted to compare the distributions of the categorical variables between Nullip vs PPBC groups, and the paired comparisons between subgroups. For all tables and dot plot graphs, continuous outcomes are expressed as the mean ± SEM, and categorical outcomes are presented as frequencies and the percentages. For Kaplan–Meier analysis, Recurrence Free Survival (RFS) is defined as the time from the date of diagnosis to the date of local recurrence, reginal recurrence or the last date of follow-up, whichever comes first. The nonparametric log rank test was conducted to compare the recurrence-free survival curves of SEMA7A low vs high groups for PPBC patients and for Nulliparous patients separately. The optimal cutoff level of SEMA7A were obtained using R package ‘survminer’^21^. The cutoff level of SEMA7A is set to 36.1, which is slightly above the average observed in the PPBC group and can describe the bimodal feature of the distribution of the SEMA7A in this group reasonably well and is also biologically and clinically plausible. *P* values less than 0.05 were deemed statistically significant. The datasets generated during and/or analyzed during the current study are available from the corresponding author on reasonable request.

### Reporting summary

Further information on research design is available in the Nature Research Reporting Summary linked to this article.

## Supplementary information

Supplementary material

Reporting Summary

## Data Availability

The data generated and analysed during the current study are not publicly available to protect patient privacy, but will be made available from the corresponding author, Dr Virginia Borges (email address: Virginia.borges@cuanschutz.edu), on reasonable request, as described in the following metadata record: 10.6084/m9.figshare.12961706^22^. Depending on the request, researchers may need to submit an Institutional Review Board (IRB) application.
