# Supplementary material for: Semaphorin 7a is a biomarker for recurrence in postpartum breast cancer
Source: NPJ Breast Cancer. 2020 Oct 19;6:56. doi: 10.1038/s41523-020-00198-1 (PMC7572422; doi:10.1038/s41523-020-00198-1)
Supplement: Supplementary file 1 — Supplementary material [file 41523_2020_198_MOESM1_ESM.pdf]

		Nullip	PPBC	p	test
**n (%)**		47	66		
**Age at Diagnosis (mean (SD))**		36.64 (±5.45)	35.85 (±5.04)	0.429	
**Race**	White	38 (80.8)	49 (74.2)	0.261	exact
	Black	0	4 (6.0)		
	Hispanic	3 (6.4)	1 (1.5)		
	Other	1 (2.1)	2 (3.0)		
	Unknown	5 (10.6)	10 15.2)		
**Tumor Histologic Subtype**	Ductal	42 (89.4)	52 (78.8)	0.406	exact
	Ductal + lobular	1 (2.1)	1 (1.5)		
	Inflammatory	2 (4.3)	2 (3.0)		
	Lobular	2 (4.3)	6 (9.1)		
	Missing	0 (0.0)	1 (1.5)		
	Other	0 (0.0)	4 (6.1)		
**Stage**	I	6 (12.8)	7 (10.6)	0.468	exact
	II	35 (74.5)	43 (65.2)		
	III	5 (10.6)	11 (16.7)		
	IV	1 (2.1)	5 (7.6)		
**LN**	Negative	16 (34.0)	22 (33.3)	0.436	exact
	Positive	28 (59.6)	43 (65.2)		
	Unknown	3 (6.4)	1 (1.5)		
**Estrogen Receptor Status**	ER-	16 (34.0)	21 (31.8)	0.841	exact
	ER+	31 (66.0)	45 (68.2)		
**Progesterone Receptor Status**	Missing	0 (0.0)	1 (1.5)	0.824	exact
	PR-	20 (42.6)	25 (37.9)		
	PR+	27 (57.4)	40 (60.6)		
**Tumor Biologic Subtype**	Luminal A (ER+/PR+/Her2-)	10 (21.3)	11 (16.7)	0.897	exact
	Luminal B (ER+/ PR+,-/ Her2 + or Her2-, and high Ki67)	21 (44.7)	33 (50.0)		
	Luminal Unknown (ER + but unable to distinguish Luminal A and B)	1 (2.1)	1 (1.5)		
	Triple Negative (ER-/PR-/HER2-)	15 (31.9)	21 (31.8)		
**Tumor Pathologic Grade**	Grade I	3 (6.4)	6 (9.1)	0.922	exact
	Grade II	15 (31.9)	20 (30.3)		
	Grade III	26 (55.3)	34 (51.5)		
	Missing	3 (6.4)	6 (9.1)		
**LVI**	Absent	18 (38.3)	24 (36.4)	0.757	exact
	Present	17 (36.2)	28 (42.4)		
	Unknown	12 (25.5)	14 (21.2)		

Supplementary Table 1: Clinical characteristics of Nulliparous and PPBC patients.

		PPBC
		66
**Gravidity (G) (%)**		
	1	12 (18.2)
	2	29 (43.9)
	3	13 (19.7)
	4	7 (10.6)
	5	2 (3.0)
	Missing	3 (4.5)
**Parity (P) (%)**		
	1	19 (28.8)
	2	32 (48.5)
	3	12 (18.2)
	4	2 (3.0)
	Missing	1 (1.5)
**Duration Between Last Childbirth and BC Diagnosis (Years) (%)**	0 - <=2	38 (57.6)
	>2 - <=5	28 (42.4)
	Missing	0 (0.0)

Supplementary Table 2: Reproductive history of PPBC patients.

	level	Nullip	PPBC	p	test
n (%)		47	66		
Vital Status (Per Last Contact)	Alive	33 (70.2)	51 (77.3)	0.513	exact
	Deceased	14 (29.8)	15 (22.7)		
Recurrence?	Missing	0 (0.0)	1 (1.5)	0.871	exact
	No	27 (57.4)	42 (63.6)		
	Other	3 (6.4)	4 (6.1)		
	Yes	17 (36.2)	19 (28.8)		

Supplementary Table 3: Outcomes for Nulliparous and PPBC patients in cohort.

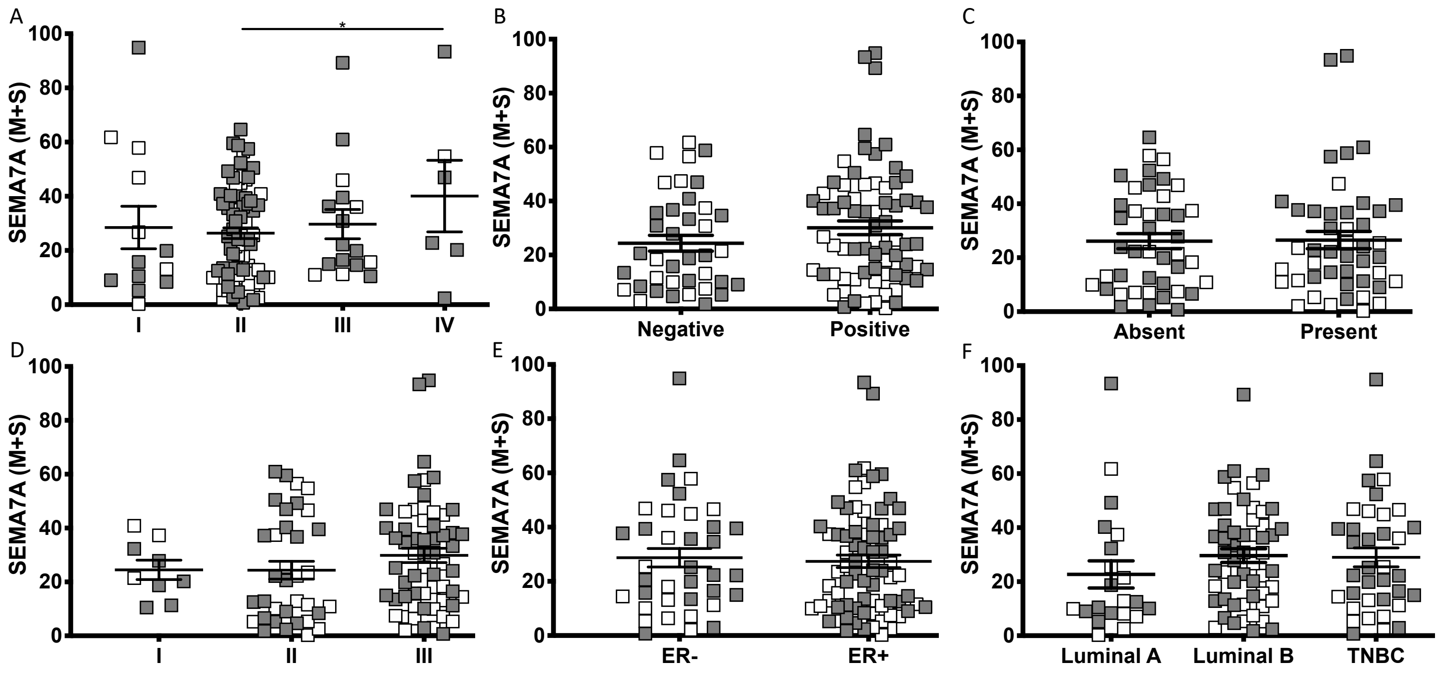

<u>Supplementary Figure 1:</u> Semaphorin 7a expression does not differ with breast cancer clinical characteristics. % SEMA7A medium + strong (M+S) staining in cohort of 113 cases with nulliparous cases denoted as white squares and postpartum cases as grey squares. A) Stage, B) Lymph node involvement, C) Lymphovascular invasion, D) Grade, E) ER status, and F) Tumor biological subtype.

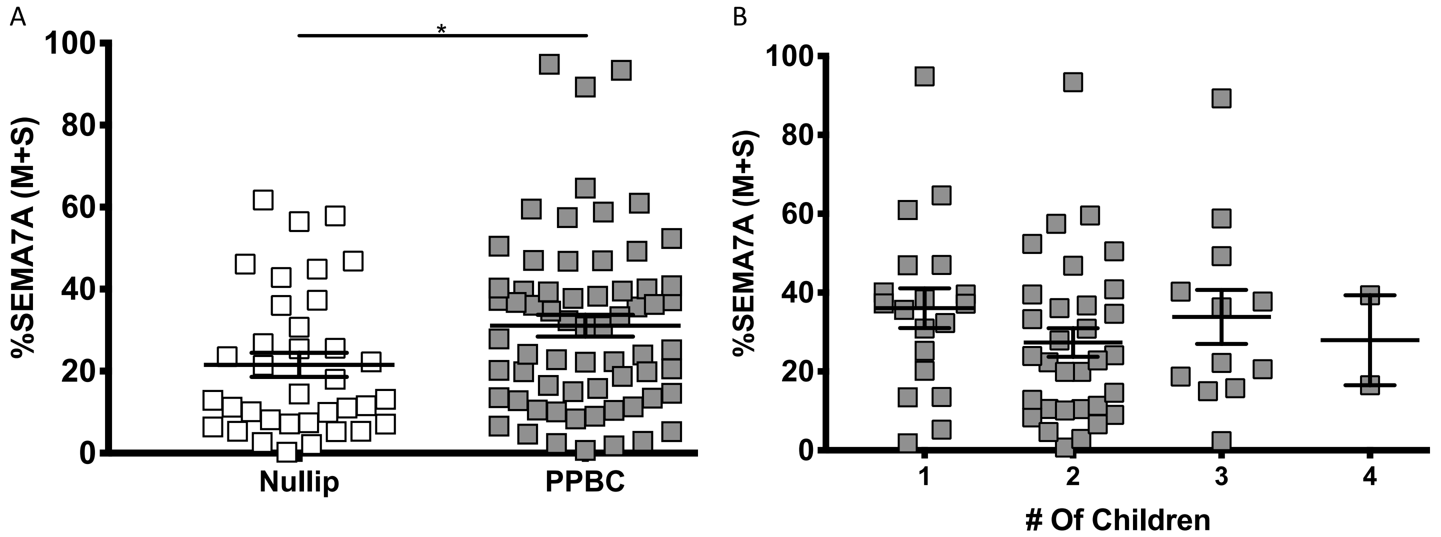

Supplementary Figure 2: A) Cases with gravidity >0 were removed in the nulliparous group. B) Semaphorin 7a expression separated by number of children.

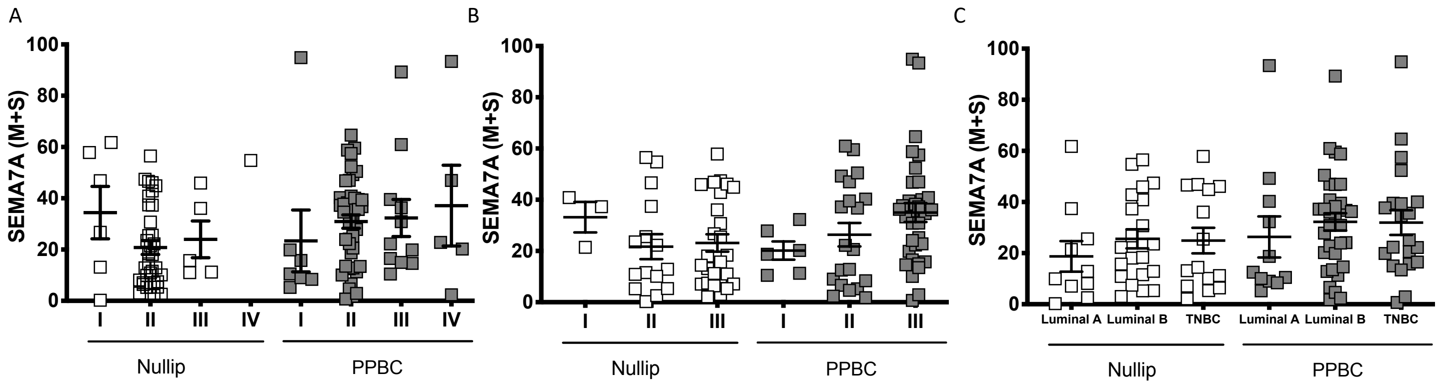

Supplementary Figure 3: SEMA7A expression in Nulliparous cases (white) and postpartum breast cancers (grey) separated by A) Stage, B) Grade, and C) Tumor Biological subtype. A-nova test.

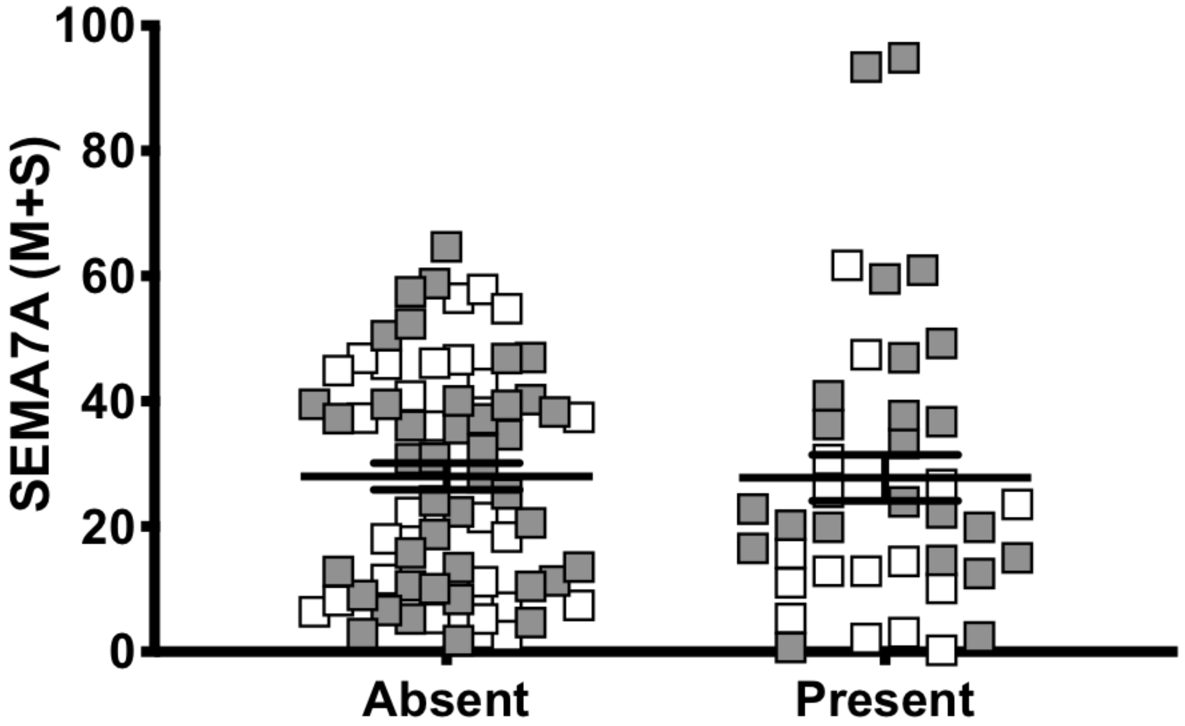

Supplementary Figure 4: Semaphorin 7a expression and the absence or presence of any recurrence (local, regional, and metastatic) in nulliparous (white) and postpartum (grey) groups.

Parity Group	PPBC (Low SEMA7a)		PPBC (High SEMA7A)	
	N= 35		N= 24	
	No. (%)		No. (%)	
**Mean age at diagnosis**	36		35	
**Age Range**	26-45		27-44	
**Histologic subtype**				
Ductal	27	77.1	21	87.5
Lobular	4	11.4	0	0.0
Ductal + Lobular	0	0.0	1	4.2
Inflammatory	1	2.9	1	4.2
Other	2	5.7	1	4.2
Missing	1	2.9		0.0
**Stage**				
0	0	0.0	0	0.0
I	6	17.1	1	4.2
II	23	65.7	20	83.3
III	6	17.1	3	12.5
IV	0	0.0	0	0.0
**Estrogen status**				
ER+	23	65.7	16	66.7
ER-	12	34.3	8	33.3
**Progesterone status**				
PR+	19	54.3	16	66.7
PR-	15	42.9	8	33.3
Missing	1	2.9	0	0.0
**Biologic subtype**				
Luminal A	8	22.9	2	8.3
Luminal B	14	40.0	14	58.3
Triple negative	12	34.3	8	33.3
Other	1	2.9	0	0.0
**Tumor Pathologic Grade**				
Grade I	5	14.3	0	0.0
Grade II	10	28.6	9	37.5
Grade III	16	45.7	14	58.3
Missing	4	11.4	1	4.2

Supplementary Table 4: Clinical characteristics of PPBC patients stratified by SEMA7A positivity.
