# Supplementary material for: Semaphorin 7a is a biomarker for recurrence in postpartum breast cancer
Source: NPJ Breast Cancer. 2020 Oct 19;6:56. doi: 10.1038/s41523-020-00198-1 (PMC7572422; doi:10.1038/s41523-020-00198-1)
Supplement: Supplementary file 2 — Reporting Summary [file 41523_2020_198_MOESM2_ESM.pdf]

nature research

Corresponding author(s): Traci R Lyons
Last updated by author(s): Aug 20, 2020

Reporting Summary

Nature Research wishes to improve the reproducibility of the work that we publish. This form provides structure for consistency and transparency in reporting. For further information on Nature Research policies, see our Editorial Policies and the Editorial Policy Checklist.

Statistics

For all statistical analyses, confirm that the following items are present in the figure legend, table legend, main text, or Methods section.

n/a	Confirmed	
☐	☒	The exact sample size (*n*) for each experimental group/condition, given as a discrete number and unit of measurement
☐	☒	A statement on whether measurements were taken from distinct samples or whether the same sample was measured repeatedly
☐	☒	The statistical test(s) used AND whether they are one- or two-sided<br>*Only common tests should be described solely by name; describe more complex techniques in the Methods section.*
☐	☒	A description of all covariates tested
☒	☐	A description of any assumptions or corrections, such as tests of normality and adjustment for multiple comparisons
☒	☐	A full description of the statistical parameters including central tendency (e.g. means) or other basic estimates (e.g. regression coefficient) AND variation (e.g. standard deviation) or associated estimates of uncertainty (e.g. confidence intervals)
☐	☒	For null hypothesis testing, the test statistic (e.g. *F*, *t*, *r*) with confidence intervals, effect sizes, degrees of freedom and *P* value noted<br>*Give P values as exact values whenever suitable.*
☒	☐	For Bayesian analysis, information on the choice of priors and Markov chain Monte Carlo settings
☒	☐	For hierarchical and complex designs, identification of the appropriate level for tests and full reporting of outcomes
☒	☐	Estimates of effect sizes (e.g. Cohen's *d*, Pearson's *r*), indicating how they were calculated

Our web collection on statistics for biologists contains articles on many of the points above.

Software and code

Policy information about availability of computer code

Data collection	R package 'survminer' was used for Kaplan Meier analysis.
Data analysis	Prism was utilized for all t-test analyses

For manuscripts utilizing custom algorithms or software that are central to the research but not yet described in published literature, software must be made available to editors and reviewers. We strongly encourage code deposition in a community repository (e.g. GitHub). See the Nature Research guidelines for submitting code & software for further information.

Data

Policy information about availability of data

All manuscripts must include a data availability statement. This statement should provide the following information, where applicable:
- Accession codes, unique identifiers, or web links for publicly available datasets
- A list of figures that have associated raw data
- A description of any restrictions on data availability

NA

Field-specific reporting

Please select the one below that is the best fit for your research. If you are not sure, read the appropriate sections before making your selection.

☒ Life sciences ☐ Behavioural & social sciences ☐ Ecological, evolutionary & environmental sciences

For a reference copy of the document with all sections, see nature.com/documents/nr-reporting-summary-flat.pdf

Life sciences study design

All studies must disclose on these points even when the disclosure is negative.

Sample size	We included 113 cases from our Colorado Young Women's Breast Cancer Cohort where we published an increased risk for metastatic recurrence in PPBC. There were 47 nulliparous patients and 66 PPBC patients that had sufficient tissue to make it into the study
Data exclusions	No data were excluded.
Replication	The is a small pilot study presented as a case report. We are in the process of expanding our analysis to a larger cohort where we expect our results will be replicated. Additionally, our results replicate our previous findings that SEMA7A is associated with poor prognosis.
Randomization	Randomization is not relevant to our study because patients were selected based on parity status to test our hypothesis.
Blinding	All investigators were blinded to study group during data analysis.

Reporting for specific materials, systems and methods

We require information from authors about some types of materials, experimental systems and methods used in many studies. Here, indicate whether each material, system or method listed is relevant to your study. If you are not sure if a list item applies to your research, read the appropriate section before selecting a response.

Materials & experimental systems

n/a	Involved in the study	
☐	☒	Antibodies
☒	☐	Eukaryotic cell lines
☒	☐	Palaeontology and archaeology
☒	☐	Animals and other organisms
☐	☒	Human research participants
☒	☐	Clinical data
☒	☐	Dual use research of concern

Methods

n/a	Involved in the study	
☒	☐	ChIP-seq
☒	☐	Flow cytometry
☒	☐	MRI-based neuroimaging

Antibodies

Antibodies used	SEMA7A clone C-6, Santa Cruz
Validation	We have validated this antibody using control and knockdown human cell lines by immunoblot and immunohistochemical methods (see Black et al Oncogene 2016).

Human research participants

Policy information about studies involving human research participants

Population characteristics	The Colorado Young Women's Breast Cancer Cohort includes a large data base of breast cancer cases with a subset having tissue obtained from clinically indicated core biopsies or surgical procedures. For this database, all clinical data were originally collected from the patient's medical record with outcomes obtained from our tumor registry q 6 months. Pathologic characteristics are collected directly from the original pathology reports. Race is a self-reported variable obtained from the patient at the time of first visit to the institution. Eligibility for the main protocols include: ≤45 years of age at diagnosis, availability of parity data, acceptance of recommended treatment for curative intent cases, and follow-up for breast cancer recurrence. The data are stored in RedCap™. To compare the SEMA7A levels between the patients' groups of interest, we used a matched design with parity being the main criteria of interest, as follows: nulliparous with and without breast cancer recurrence compared to PPBC with and without breast cancer recurrence, as our main experimental groups. All patients were premenopausal at the time of diagnosis. Nulliparous cases were defined as never pregnant (G0P0) or without completed childbirth (GXP0), where X may be any number, as we have previously published no difference in outcomes among women with prior incomplete pregnancies (GXP0, X=1 or more) and breast cancer recurrence7. PPBC cases were defined as having completed at least one childbirth (GXPX), where X=1 or more, and having received a cancer diagnosis

	within five years of parturition. Given the strong influence biologic subtype has on breast cancer recurrence, we intentionally aimed to select approximately 70% to be ER positive and 30% to be TNBC (ER/PR/Her2 negative) between nulliparous and PPBC cases. Only Her2 negative cases were included for simplicity in this pilot study to eliminate confounding variables of crosstalk from overlapping signaling pathways. Lastly, we selected a random sample of the identified cases that had available tumor sections which included normal-adjacent tissue. Additional data on clinically significant variables were available as follows: histologic subtype (ductal, lobular, inflammatory, other), biologic subtype (luminal A versus B determined as estrogen receptor positive and progesterone receptor positive/A or negative/B and centralized Ki-67 proliferation index testing as <14% low/A or ≥14% high/B respectively), stage (AJCC version 7 TNM criteria), lymph node involvement (LN), lymphovascular invasion (LVI), grade (from clinical pathology report), and breast cancer recurrence (absent or present, where present includes local regional recurrence, metastatic disease or both). Other status (for recurrence local/regional/ metastatic) is a combination of when a subject had either "recurrence of unknown type" or "patient stage IV never free of disease".
Recruitment	Subjects were enrolled before 2010 through a retrospective consent exempt protocol and after 2010 they were prospectively enrolled and provided informed consent for participation.
Ethics oversight	The clinical trials under which all data and human tissues were collected for this research were conducted according to Declaration of Helsinki Principles and with approval of the Colorado Multiple Institution Review Board. Subjects were enrolled before 2010 through a retrospective consent exempt protocol and after 2010 they were prospectively enrolled and provided informed consent for participation.

Note that full information on the approval of the study protocol must also be provided in the manuscript.
